# No Effects of rTMS on Performance Monitoring and Attentional Bias in Patients With Alcohol Use Disorder: A Pilot Study

**DOI:** 10.1111/adb.70100

**Published:** 2025-11-18

**Authors:** Maarten Belgers, Wiebren Markus, Federico Grasso, Martijn Arns, Philip Van Eijndhoven, Arnt Schellekens

**Affiliations:** ^1^ IrisZorg, Center for Addiction Care and Sheltered Housing Arnhem the Netherlands; ^2^ Nijmegen Institute of Scientist‐Practitioners in Addiction (NISPA) Radboud University Nijmegen Nijmegen the Netherlands; ^3^ Cooperativa Sociale P.G. Frassati Turin Italy; ^4^ Brainclinics Foundation Nijmegen the Netherlands; ^5^ Department of Psychiatry Radboud University Medical Center (Radboudumc) Nijmegen the Netherlands

**Keywords:** alcohol use disorder, attentional bias, electroencephalogram, event‐related potential, neuromarker, performance monitoring, transcranial magnetic stimulation

## Abstract

Repetitive transcranial magnetic stimulation (rTMS) presents a promising approach for treating patients with alcohol use disorder (AUD). However, its neuropsychological working mechanisms are to be elucidated. Therefore, we investigated the effect of rTMS treatment on event‐related potentials (ERPs) evoked by a flanker task and a passive picture task using electro‐encephalography, in 30 recently detoxified patients. Participants were randomly assigned to receive either 10 sessions of rTMS or sham rTMS. Measurements were made at baseline, and after 5 and 10 treatments. The results revealed no significant effect of rTMS on any of the assessed ERPs (N100, N200, and P300). These findings suggest that high‐frequency rTMS applied over the right dlPFC, in addition to treatment as usual (TAU), does not impact performance monitoring or attentional bias in recently detoxified patients with AUD. Bayesian analyses did suggest a potential effect of rTMS on N100 amplitude at frontal electrode FZ. These preliminary findings warrant further study of the effects of rTMS on ERP outcomes in AUD patients, and exploration of ERPs related to performance monitoring and attentional bias as potential neuropsychological markers for the clinical effects of rTMS. However, other neuropsychological markers, like brain network connectivity, should also be explored.

**Clinical Trial Registration:**

Registered in a trial Register (ClinicalTrials.gov Identifier: NCT01973127)f.

## Graphical abstract

We tested whether high‐frequency rTMS over right dlPFC modulates ERP markers of performance monitoring and attentional bias in 30 recently detoxified AUD patients in a randomized sham‐controlled design, assessing N100, N200, and P300 during a flanker and passive picture tasks at baseline, mid‐treatment, and post‐treatment. No significant group‐by‐time effects were observed, indicating no robust modulation of targeted ERPs. Bayesian evidence suggested a possible effect on frontal N100, supporting further exploration of neurophysiological markers of rTMS response in AUD.

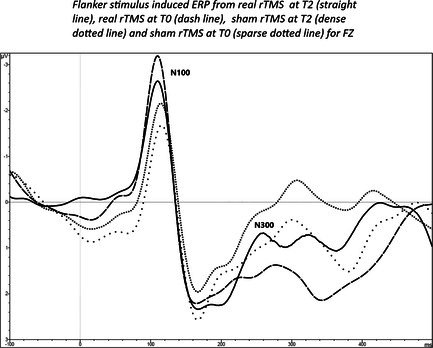

## 1 Introduction

Alcohol use disorder (AUD) is a condition characterized by the inability to limit alcohol intake, accompanied by intense cravings, and is associated with substantial distress or impairment [1]. Worldwide, 5.1% of the burden of disease and injury is attributable to use of alcohol, as measured in disability‐adjusted life years (DALYs) [2]. Given the substantial burden it poses, effective treatment options for AUD patients are imperative. With current evidence‐based treatment options (which are mainly pharmacological and psychosocial in nature) effect sizes are low to moderate, and relapse rates are as high as up to 60% within 1 year after reaching abstinence [3, 4].

Noninvasive brain stimulation offers a promising avenue for addressing AUD through a distinct mechanism compared with current treatments [5]. One such technique, repetitive transcranial magnetic stimulation (rTMS), involves the application of alternating magnetic fields via an electromagnetic coil positioned over the patient's head. However, previous research on the effectiveness of rTMS in treating patients with AUD has shown mixed outcomes [6]. A recent consensus paper emphasized the need for more studies, to assess clinical effectiveness, optimize neuromodulation parameters, and take a mechanistic approach by using objective biological metrics related to AUD [7].

Several mechanisms have been shown to be involved in AUD, including reduced cognitive control and increased cue reactivity [8]. Research indicates that alcohol adversely affects both the evaluative and regulative aspects of cognitive control [9]. Indices of these processes might be relevant measures to evaluate the effects of rTMS and further our understanding of potential working mechanisms.

Evaluative cognitive control involves action monitoring for indications of conflict, erroneous outcomes and prediction error [10]. Biological markers for action monitoring are error‐related components like the event related negativity (ERN) [11, 12, 13]. Such an event‐related brain potential (ERP) can be elicited by cognitive tasks involving quick response times and detected through analysis of an electroencephalogram (EEG) recording. The ERN, which occurs approximately 100 ms after the commission of a subjectively perceived error after stimulus processing is also called the N100 [14]. Other ERP components are the N200, N300, and N400. The later ERP components reflect more integrated processing of different brain regions. The N100 related to action monitoring most likely originates from the anterior cingulate cortex (ACC) [15]. People with SUD, including AUD show reduced N100 [16, 17]. Patients with AUD who successfully abstain from alcohol exhibit higher ACC activity in a cue‐exposure paradigm before receiving rTMS treatment compared with those who relapse leading to a more pronounced N100 after abstinence [18]. In addition to loss of control, AUD can be accompanied by increased cue‐reactivity, resulting in attentional cognitive biases and craving [19]. Cue reactivity can also be measured using ERPs, as a positive deflection on the EEG around 300 ms after cue‐stimulus onset (P300 amplitude) [20]. P300 amplitudes are enhanced in response to alcohol‐related cues as compared with neutral cues in patients with AUD, reflecting an attentional bias towards these cues [21]. One study has shown that a high frequency rTMS protocol over the dorso‐lateral prefrontal cortex (DLPFC) reduces craving and attentional bias as indexed by reduced activity in the DMN measured using fMRI [22].

Given the relatively limited number of mechanistic studies into the effects of rTMS in AUD, this study aims to investigate the effects of rTMS on cognitive control and cue‐reactivity by using EEG measurements within an ERP paradigm. Most mechanistic studies in this field have applied fMRI as a biomarker [18, 22, 23] for rTMS effects. EEG is a relatively simple technique, which is potentially easier to implement in clinical practice than more expensive fMRI measures, and has a more detailed temporal resolution than fMRI. However, to our knowledge, no studies applied ERPs in this context to date. By examining ERP's as objective metrics in a rTMS treatment protocol, we aim to gain more insight whether rTMS can reduce AUD symptoms through the restoration of evaluative cognitive control and attentional bias.

Specifically, we test the hypothesis that a high frequency rTMS stimulation protocol targeting the right dlPFC will (1) increase N100 amplitudes related to performance monitoring and (2) reduce P300 amplitudes related to attentional bias to alcohol‐related stimuli compared with a sham control‐group. Finally, we explore whether these effects accumulate over successive rTMS treatments because effectiveness of rTMS in patients with AUD seems to correlate with rTMS treatment intensity [7].

## 2 Materials and Methods

### 2.1 Study Design

The study was designed as a single‐blind randomized controlled trial and approval was obtained from the Medical Ethical Committee of the Radboud University Medical Centre (protocol no. NL46974.091.13), and the trial was preregistered in a trial registry (ClinicalTrials.gov Identifier: NCT01973127). Clinical findings, showing decreased craving in alcohol and decreased alcohol intake after rTMS treatment compared with sham rTMS, have been published previously [24].

### 2.2 Study Sample

Eligible patients diagnosed with AUD were recruited between 2015 and 2019 from two addiction care centers (IrisZorg and Novadic‐Kentron) and the Radboud University Medical Centre in the Netherlands. Of 37 individuals screened for eligibility, 34 were included and randomized, with 3 unreachable after initial contact. After baseline assessments, four individuals withdrew their consent to participate without starting treatment. A total of 30 participants were included in the analyses.

Inclusion criteria comprised the following: (1) meeting DSM‐5 criteria for AUD as the primary diagnosis assessed via the Structured Clinical Interview for DSM‐5 Disorders—Clinician Version (SCID‐5‐CV) [25], (the use of other substances was not an exclusion criterion); (2) age between 20 and 65 years; (3) successful recent (< 6 weeks) completion of inpatient alcohol detoxification, and (4) written informed consent. Exclusion criteria included the following: (1) any severe psychiatric condition interfering with participation of the study or treatment as usual (TAU) (e.g., acute psychosis, acute suicidality, or severe cognitive disabilities); (2) contraindications for standard rTMS (e.g., history of epilepsy, ferromagnetic implants in the head, neurosurgical operations, or a pacemaker implant); (3) use of medications known to significantly lower the seizure threshold (e.g., clozapine, pethidine, and aminophylline), and (4) other factors rendering study procedures infeasible (notably intellectual disabilities, major somatic disabilities, or insufficient proficiency in Dutch language). Based on studies with ERP's as outcome measurements of Noda et al. [26], Capotosto et al. [27], and Jing et al. [28], with a Cohen's *f* of 0.4 (moderate effect size), a power of 80%, a *p* value of 0.05, 5 measurements in two groups, and a correlation of 0.5 between repeated measures, we a priori calculated a total sample size needed of 14 patients in two arms.

### 2.3 Treatment

#### 2.3.1 Treatment as Usual

All participants received TAU throughout the entire duration of the study, including the follow‐up period at one of the participating addiction care centers. TAU involved both inpatient and outpatient cognitive behavioral therapy (CBT) or community reinforcement approach therapy (CRA) and/or anti‐craving medication.

#### 2.3.2 rTMS

rTMS was administered with a 70‐mm figure‐of‐eight coil (Air Film coil) and a Magstim Rapid2 stimulator [29]. Initially, the target for the rTMS coil was identified as the F4 location over the right dlPFC, following the international 10–20 system for electroencephalography [30]. Once F4 was determined, it was marked on a cap positioned on the participant's head relative to anatomical landmarks of the skull (nasion–inion) to ensure accurate targeting during subsequent treatment sessions. Subsequently, the resting motor threshold (MT), defined as the intensity at which motor neurons elicit muscular contraction in the contralateral hand in at least 5 out of 10 trials, was established by delivering single pulses of TMS with gradually increasing intensity over the right motor cortex. During each rTMS treatment session, participants received sixty 10‐Hz trains lasting 5 s each, with an inter‐train interval of 25 s, delivered at 110% of MT intensity, resulting in 3000 pulses per session (totaling 30 min of treatment time) and 30 000 pulses over the entire study duration. The total of 30 000 pulses remains well below the threshold associated with increased risk of side effects [31].

#### 2.3.3 Sham rTMS

Subjects in the sham group were stimulated with the coil tilted at 90° with only the edge of the coil touching the scalp [32]. This placement directs the magnetic field away from the brain while maintaining clicking sounds and muscular twitching while only causing superficial neural stimulation [33, 34]. The investigator (first author) was not blinded to the treatment modality. Both the real and sham groups received hearing plugs during treatment sessions.

### 2.4 Measurements

#### 2.4.1 Sample and Treatment Characteristics

The baseline assessment included the following variables: age, sex, handedness, estimate of IQ (assessed using the Dutch version of the Adult Reading Test (NLV) [35]), years of education, and the use of anti‐craving, antidepressant, and antipsychotic medications at baseline (yes/no). Additionally, the duration of AUD in years, the number of previous AUD treatments, and the presence of psychiatric disorders and other substance use disorders were assessed using the Mini‐International Neuropsychiatric Interview (MINI) [36].

#### 2.4.2 Outcome Measurements

##### 2.4.2.1 Flanker Task

Participants were presented with a modified perceptual flanker task [37] to evoke ERPs related to performance monitoring, and were required to respond with either their left or right index finger to the central letter (H or S) of a congruent (HHHHH or SSSSS) or incongruent (HHSHH or SSHSS) letter string. Initially, a fixation point appeared for 100 ms, followed by the stimulus 300 ms later (stimulus duration: 100 ms). Subsequently, the screen remained blank for 900 ms, after which a visual feedback stimulus was displayed for 1000 ms. The subsequent trial commenced after a 1000‐ms inter‐trial interval. The visual feedback was provided through a yellow, blue, or red rectangle indicating whether the response was correct, incorrect, or too late, respectively. Participants were instructed to respond as quickly as possible to avoid receiving feedback indicating their response was too slow, based on a fixed preset reaction‐time (RT) deadline of 500 ms. Following verbal instructions, participants familiarized themselves with the task during a practice block of 60 trials. The experimental phase comprised two times five blocks of 100 trials, with a self‐paced pause halfway through each block. Total task duration was between 20 and 35 min.

##### 2.4.2.2 Passive Picture Viewing Task

To assess alcohol related attentional bias, a passive picture viewing task was used. The images utilized in this task were sourced from The Geneva Appetitive Alcohol Pictures (GAAP) [38], a normative database of affective alcohol‐related pictures, provides a large number of stimuli for investigators who conduct research on alcohol. Sixty alcohol‐related pictures (beverages, drinking‐related behaviors, alcohol‐related cues) and 60 neutral images (sourced from the Geneva affective picture database [39]) were standardized to a size of 12° × 9° and presented at random for 5 s (with a blank interval between pictures of 5 s) on a monitor. The normative valence and arousal ratings of these affective and neutral images have been previously documented [38, 39]. Total task duration was 40 min.

##### 2.4.2.3 EEG Measurements

The EEG was assessed using 28 Ag/AgCl scalp electrodes affixed in an elastic electrode cap (ActiCap, Brain Products, Munich, Germany). All electrodes were referenced to the left mastoid, the ground was placed on the left side of the nose. EEG signals were recorded and digitized using an EEG amplifier (QuickAmp, Brain Products GmbH, Munich) at a sample rate of 1000 Hz without online filtering and with BrainVision EEG‐recording software (Brain Vision Recorder software, Brain Products GmbH, Munich, Germany). The recorded electrodes were positioned at five midline (FZ, FCZ, CZ, PZ, OZ) and 23 lateral (F3, F4, F7, F8, FC1, FC2, FC5, FC6, C3, C4, T7, T8, CP1, CP2, CP5, CP6, TP8, P3, P4, P7, P8, O1, O2) locations following the international 10–10 system [40]. Additionally, the vertical electro‐oculogram (EOG) was recorded bipolarly from electrodes positioned placed 3 mm above the left eyebrows and 1.5 cm below the left bottom eyelid. The horizontal EOG was similarly recorded from electrodes located lateral 1.5 cm lateral to the outer canthus of each eye, respectively. All electrode impedances were kept below 5 kΩ at the start of the recording session and were monitored during the test session.

### 2.5 Procedure

**FIGURE 1:**
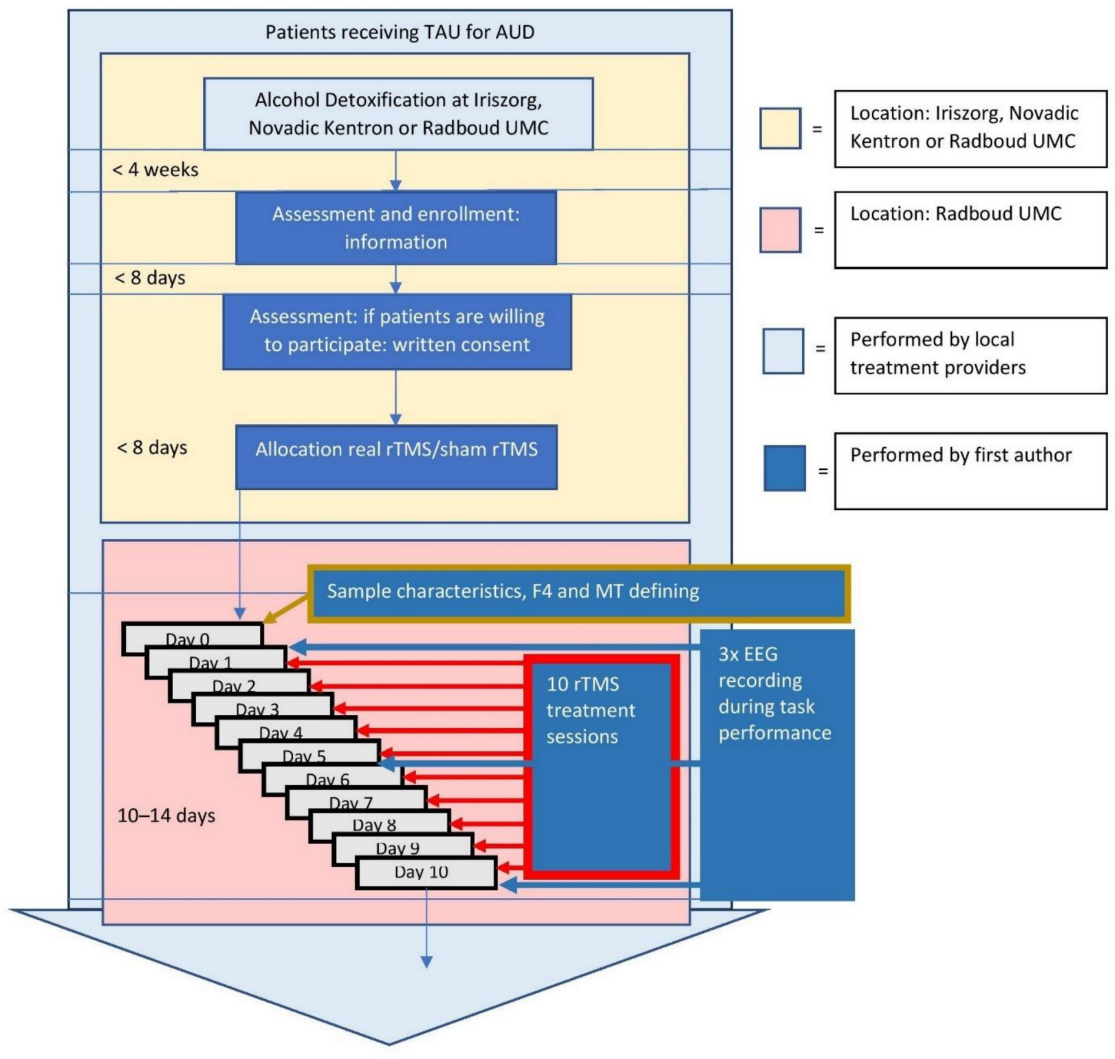
Schematic overview of the study procedure. AUD = alcohol use disorder; rTMS = repetitive transcranial magnetic stimulation; TAU = treatment as usual.

Details of recruitment were published elsewhere [24]. Patients were recruited form addiction care centers and Radboud University Medical Centre. After randomly being allocated to one of two groups, participants received accordingly 10 treatments of real or sham rTMS over 10–14 days. Task and EEG recordings were performed at baseline and at 5 and 10 treatment sessions (Figure 1).

### 2.6 Analyses

The Statistical Package for Social Sciences (SPSS) version 28 and Jeffreys's Amazing Statistics Program (JASP) version 0.95.0 were used to analyze the data [41, 42]. *p* values < 0.05 were considered significant.

#### 2.6.1 Sample and Treatment Characteristics and Outcome Variables at Baseline

Baseline sample characteristics were summarized using descriptive statistics and compared between groups. For categorical variables, comparisons were performed with a *χ* ^2^ test while Fisher's exact tests were used in case the expected cell counts were less than five. A two‐sample *t* test was used for continuous variables in case normality was met (Kolmogorov–Smirnov test); otherwise, the non‐parametric Mann–Whitney *U* test was applied.

#### 2.6.2 EEG Analysis

EEG data were analyzed using BrainVision Analyzer Version 2.2 [43]. Electro‐oculogram (EOG) correction was applied in line with Gratton [44]. All channels were referenced to an average of all recorded channels. A high‐pass filter of 1 Hz and a low‐pass filter with a cutoff frequency of 30 Hz was applied to the data. Baseline correction was performed by subtracting the average amplitude of the 100‐ms pre‐stimulus interval. The EEG data were averaged for each participant. Peak data were semi‐automatic retrieved in the software on the averages per participant.

#### 2.6.3 Statistical Analysis

The amplitude of the stimulus‐locked N100 component was characterized as the maximum negative deflection observed within the 50–150 ms post‐stimulus interval. The N200 component was defined by the most negative amplitude occurring between 150 and 250 ms following stimulus onset while the N300 component was identified by the most negative deflection occurring between 250 and 350 ms. The amplitude of the P300 component was characterized as the largest positive deflection observed within the 300–500 ms post‐stimulus interval.

We analyzed the various event‐related potentials (ERPs) using a general linear model with repeated measures analysis [41]. The factors included time (with three levels: baseline, post‐five sessions, and post‐ten sessions) as a within‐subject factor and condition (real rTMS or sham) as a between‐subject factor. These analyses were conducted separately for three electrodes (Fz, Cz, and Pz) (see Figure S1). These electrodes were chosen based on their frequent use in ERP studies, as the amplitudes of different ERP components are typically largest at these sites, consistent with the literature and our findings [45, 46].

We analyzed the stimulus‐locked N100 and N200 component with a Bayesian repeated measure ANOVA, with the three mentioned time factors as within‐subject factors and condition (real‐sham) as between‐subject factor for electrodes Fz, Cz, and Pz separately and calculated the Bayesian factor (BF10).

#### 2.6.4 Behavioral Results Analysis

A general linear model with a repeated measures analysis was performed on individual averages for reaction times (RTs) on correct and incorrect answers and answers that were too late for the three time points. This was also used for analyzing accuracy of reaction performance.

## 3 Results

All participants had six or more DSM‐5 AUD criteria, indicating severe AUD. No significant differences in baseline characteristics were found between groups, except for PTSD (Fisher's exact test *p* = 0.007), with more comorbid PTSD in the sham group. All patients were right‐handed. One participant in the TAU group consumed a small amount of alcohol on a single occasion between intake and the first treatment session. Although this did not constitute an exclusion criterion, it meant that the TAU group was not fully abstinent at baseline. For baseline sample characteristics see Table S1. The clinical results of this study were published elsewhere, in which it was shown that rTMS leads to better clinical outcomes in terms of craving (Wilks' Λ = 0.348, *F* [12, 17] = 2.654, *p* = 0.032) and alcohol use group (Λ = 0.46, *F* [5, 24] = 5.6, *p* = 0.001), in comparison with sham treatment during a follow‐up period of 12 months. For more detailed information about the clinical outcomes, see Belgers et al. [24].

### 3.1 Behavioral Outcomes of Flanker Task

The repeated measures ANOVA for correct, incorrect trials and “too late” (no response 500 ms after stimulus) trials showed that there were no differences between the rTMS and sham group on the reaction times at any time point (Figure S2).

The repeated measures ANOVA for the accuracy of correct and incorrect trials showed no difference between rTMS and sham group with time as a within‐subjects factor (Wilks' Λ = 0.946, *F* (2, 27) = 0.772, *p* = 0.472) (Figure S3).

### 3.2 Stimulus‐Locked ERN Flanker Task

Averaging segments after response showed a N100 most prominent at electrode FZ, both N100 and N300 at electrode CZ and N300 most prominent at PZ. A N200 ERP could not be identified at either electrode, a clear N100 was absent at electrode Pz (Figure S4).

Repeated measures ANOVA's for ERP's on electrodes Fz, Cz, and Pz showed no difference between rTMS and sham group with time as a within‐subjects factor (see Table 1 and Figure S5), indicating no effect of rTMS on performance monitoring as marked by ERP N100 and N300. A repeated measure ANOVA sub analysis with the three electrodes Fz, Cz, and Pz as dependent variables showed no differences between group with time as a within‐subject factor (Wilks' Λ = 0.937, *F* (4, 25) = 0.422, *p* = 0.791). Sub‐analysis with PTSD or medication use as a covariate did not alter these results.

**TABLE 1:** Effects between rTMS and sham rTMS group with time (T0, T1, and T2) as within‐subject factor in flanker task.

ERP	Electrode	Wilks' Λ	*F*	*df*	*p*
N100	FZ	0.831	2.753	2, 27	0.082
	CZ	0.875	1.928	2, 27	0.165
	PZ	0.813	3.106	2, 27	0.061
N300	FZ	0.868	2.047	2, 27	0.149
	CZ	0.951	0.695	2, 27	0.508
	PZ	0.913	1.285	2, 27	0.293

A Bayesian repeated measure ANOVA only showed an effect of rTMS on the N100 at FZ (Bf10 = 1.089, error% 4.96), and no other effects (Bf10 between 0.33 and 1; Figures S6 and S7).

### 3.3 Passive Picture Task

Averaging segments after stimulus showed a N100 between 50 and 150 ms and a P300 starting between 250 and 350 ms (Figure S8).

Repeated measures ANOVA's for ERP's (N100 and P300) on electrodes Fz, Cz, and Pz showed no difference between rTMS and sham group with time as a within‐subjects factor (see Table 2 and Figure S9), indicating no effect of rTMS on attentional bias as marked by ERP N100 and P300.

**TABLE 2:** Effects between rTMS and sham rTMS group with time (T0, T1, and T2) as within‐subject factor for alcohol related pictures in passive picture task.

ERP	Electrode	Wilks' Λ	*F*	*df*	*p*
N100	FZ	0.979	2.151	2, 27	0.136
	CZ	0.895	1.590	2, 27	0.320
	PZ	0.920	1.180	2, 27	0.323
P300	FZ	0.887	1.728	2, 27	0.197
	CZ	0.940	0.861	2, 27	0.434
	PZ	0.923	1.120	2, 27	0.341

No differences between rTMS a sham rTMS group in ERP's related to the different picture groups were observed (Figure S10). Performing a repeated measures subanalysis on the difference between the P300 amplitude in alcohol related images and non‐alcohol related images, did not show a difference between groups by time (Wilks' Λ = 0.956, *F* (2, 27) = 0.623, *p* = 0.544), indicating no effect of rTMS on attention regardless of cues.

## 4 Discussion

This study explored the impact of rTMS on performance monitoring and attentional bias towards alcohol cues among recently detoxified individuals with AUD in a single‐blind randomized controlled trial. The study applied a rTMS treatment protocol targeting the right dlPFC over a span of 10 days. Although the rTMS treatment was beneficial in terms of clinical outcomes, this study revealed no significant differential effect of the rTMS intervention on ERPs associated with performance monitoring and attentional cue reactivity in comparison with sham rTMS.

This outcome contradicts the initial hypothesis that rTMS would enhance performance monitoring and reduce attentional bias towards alcohol‐related cues, thereby decreasing alcohol consumption and craving. Because in this study we did observe reductions in craving and alcohol consumption in the real rTMS group compared with the sham group [24], this suggests that either the ERP measurements used in this study are not reliable biomarkers for rTMS effects on craving or alcohol use, and/or rTMS exerts its effects through other neuropsychological mechanisms than performance monitoring or attentional bias, as assessed here. Bayesian analyses showed some evidence for an effect of rTMS on restoring performance monitoring, in line with the observed trend in the other analyses, suggesting this sample might have been underpowered. Based on our findings, we calculated that a sample size of 32 in both arms could potentially lead to significant findings of rTMS on performance monitoring in AUD patients (boundary BF10 = 10; Figure S11).

On the clinical outcomes, no differences were observed between groups immediately following the 10‐day treatment period, which aligns with the lack of EEG measurement findings at that timepoint. However, at timepoint 1 and 3 months after treatment, reduced alcohol craving and consumption were observed. This lack of ERP findings could indicate that these measures are unreliable for the assessment of rTMS effects. Though ERPs were clearly observed, suggesting our measures were sound, the lack of a healthy control group hinders conclusions about alterations in ERP measures in this AUD population. If we selected a group of AUD patients without ERP alterations as compared with controls, it is unlikely to find clear rTMS effects. Although ERP's were clearly observed, these ERP's exhibited slightly reduced slopes and amplitudes compared with similar studies with populations with less severe addictions using the exact same measurements and analyzing methods [45]. This could be related to for instance inattention in our population, influence of medication or (lasting) effects of the effects of long‐term excessive alcohol use.

Furthermore, surprisingly in the cue‐exposure paradigm, no differences were observed between alcohol‐related and neutral pictures. This is in contrast with literature indicating cue‐reactivity as a hallmark of addictive disorders [47]. Methodological limitations, such as diminished attention, lack of personalized cue stimuli, or insufficient engagement with images, may have contributed to the absence of cue‐reactivity findings. The alcohol‐related pictures in our study were not matched to the individual alcohol preferences of the participants. Literature suggests that this matching is important for inducing neural responses in the dlPFC [48]. However, several other studies also failed to identify cue‐reactivity in patients with SUD/AUD [49].

Besides EEG markers, fMRI could provide other biological markers of craving in patients with AUD. A recent study employing machine learning on fMRI data identified a multivariate pattern of brain activation, termed the “neurobiological craving signature (NCS),” which predicts cue‐induced craving. This NCS encompasses areas such as the striatum, prefrontal cortex, insula, amygdala, parietal, occipital, and cerebellar regions. In other studies, proposed neuromarkers for craving are also associated with various brain regions and the functional connectivity of networks such as the default mode network (DMN), attentional network, and sensory and motor‐related networks [50, 51]. These findings align with the theoretical concept of craving as a multifaceted phenomenon, involving cognitive, emotional, sensory, and physiological components that are not confined to specific brain areas but rather involve the functioning of interconnected networks [52, 53]. From this perspective, the N100, N300, and P300 components appear to be too limited or non‐specific to serve as reliable neuromarkers for the response to rTMS in patients with AUD.

rTMS may also exert its clinical effects through other mechanisms then by enhancing performance control or reducing attentional bias. A recent meta‐analysis evaluating rTMS effects on symptom domains found large effects on craving, while the effects on cognitive control and memory were small, and the effect on attention was not significant [54]. In studies on the effects of rTMS in patients with major depressive disorder (MDD), recent systematic reviews indicate that rTMS effectiveness is related to restoring network connectivity, particularly between the DMN, the executive control network, and the salience network [55]. Neurobiological alterations in brain network connectivity play a prominent role in the development of addiction [56]. This may explain the absence of significant effects when focusing on top‐down processes and suggests that investigating factors such as network connectivity could yield more informative insights. Future studies could elucidate whether these findings in MDD patients can be related to the mechanisms of rTMS in patients with AUD.

Another explanation for the observed lack of an rTMS effect on ERPs could be related to the timeframe between the baseline measurement and the two follow‐up measurements, which may have been too short. Existing literature correlating ERPs with clinical outcomes such as alcohol use and craving typically involves participants with prolonged abstinence (several months) [57]. Additionally, the effects of rTMS require time to manifest in clinical outcomes, with delayed effects persisting for weeks to months [58]. This suggests that both clinical and ERP effects of rTMS (in combination with alcohol abstinence) may become more pronounced after a longer duration. A recent meta‐analysis revealed that studies focusing on the left dlPFC in patients with SUD showed a reduction in craving, unlike those targeting the right dlPFC [59]. The neurobiological mechanisms of brain lateralization in SUD remain poorly understood, implying the possibility that (the effects of rTMS on) performance monitoring and attentional bias as measured with ERP also shows lateralization.

At the outset of this study in 2014, the right DLPFC was regarded as the most promising target for rTMS in AUD [60]. However, evolving evidence favors stimulation of the left DLPFC or the medial PFC [59, 61]. Our negative findings may thus be related to targeting the right instead of the left DLPFC. Future studies should assess whether stimulation of other, more optimal targets in AUD populations, may show clear effects on EEG‐related indicators of brain function. The discrepancy between our clinical and EEG findings may indicate that clinical non‐responders attenuated any observable EEG effects, which might only become detectable in larger samples. Future studies with larger samples could also assess the relationship between changes in ERP measures and clinical effectiveness of rTMS interventions.

The findings in this study should further be seen in the light of some study limitation. First, compared with rTMS literature in MDD, the number of treatments in our study was low. In MDD, treatment duration is most often 4 to 8 weeks. It could be speculated that longer duration of rTMS treatment could have resulted in effects of rTMS on ERP measures [55]. Second, literature indicates that P300 amplitudes and mismatch negativity (MMN) can be affected by benzodiazepines and antidepressants [62]. Indeed, 50%–72% of participants (sham vs. rTMS, respectively) used anti‐depressants, whereas 19%–44% of participants (sham vs. rTMS, respectively) used benzodiazepines. Consequently, our measurements may have been influenced by the use of these medications. Third, we acquired some ERP data in a passive manner. A P300 amplitude yielded by a passive oddball is considerably smaller than one provoked by an active oddball paradigm, which could explain the lack of observed differences. Lastly, the small sample size, the single‐blinded study design, and potential uncertainty regarding the consistent anatomical placement of the rTMS coil could have further contributed to a lower chance of finding rTMS effects on the ERP measures.

## 5 Conclusions

This small‐scale randomized trial demonstrates no effect of high‐frequency rTMS administered over the right dlPFC on performance monitoring and attentional bias as measured by ERP's in recently detoxified patients with AUD. This finding contrasts with the clinical results of this study, where reductions in craving and alcohol consumption were observed [24]. However, based on these preliminary findings, we conclude that a larger sample size could be required to detect potential rTMS effects on EEG outcome measures in AUD patients. Furthermore, clinical effects of rTMS in AUD may not work mainly through changes in performance monitoring or attentional bias. Based on literature regarding rTMS effects on both AUD and MDD, it can be hypothesized that the effect of rTMS in AUD may be more related to changes in network connectivity, suggesting the need for other neuropsychological or neuroimaging markers.

## Author Contributions

Conceptualization, A.S., M.B., and W.M.; methodology, W.M. and M.B.; validation, A.S. and M.B.; formal analysis, M.B. and M.A.; investigation, M.B.; data curation, M.B.; writing and original draft preparation, M.B. F.G.; writing, review, and editing, M. B, W.M., P.v.E., M. A, and A.S.; visualization, M.B.; supervision, W.M., M.A., and A.S.; project administration, M.B. All authors have read and agreed to the published version of the manuscript.

## Ethics Statement

The study was conducted in accordance with the Declaration of Helsinki and approved by the Medical Ethical Committee of the Radboud University Medical Centre (protocol nr. NL46974.091.13).

## Consent

Informed consent was obtained from all subjects involved in the study.

## Conflicts of Interest

The authors declare no conflicts of interest.

## Permission to Reproduce Material From Other Sources

The authors have nothing to report.

## Supporting information


**Table S1** *Baseline Sample Characteristics*.
**Figure S1.** *Location of Fz, Cz, Pz of the EEG and rTMS target location on the right dLPFC*.
**Figure S2.** *Flanker task reaction times for correct responses (left graph) incorrect responses (middle graph) and too late responses (right graph) at start (T0), halfway (T1) and end (T2) of treatment.*
**Figure S3.** *Reaction accuracy for correct responses (left graph) incorrect responses (right graph) at start (T0), halfway (T1) and end (T2) of treatment.*
**Figure S4a.** *Flanker stimulus induced ERP from real rTMS at T2 (straight line), real rTMS at T0 (dash line), sham rTMS at T2 (dense dotted line) and sham rTMS at T0 (sparse dotted line) for FZ.*
**Figure S4b.** *Flanker stimulus induced ERP from real rTMS at T2 (straight line), real rTMS at T0 (dash line), sham rTMS at T2 (dense dotted line) and sham rTMS at T0 (sparse dotted line) for CZ.*
**Figure S4c.** *Flanker stimulus induced ERP from real rTMS at T2 (straight line), real rTMS at T0 (dash line), sham rTMS at T2 (dense dotted line) and sham rTMS at T0 (sparse dotted line) for PZ*.
**Figure S5.** *Repeated measures ANOVA N100 at electrode Fz at the three time points*.
**Figure S6.** Bayesian plot of Flanker‐stimulus locked N100 at FZ for both real and sham group.
**Figure S7.** Bayesian plot of Flanker‐stimulus locked N100 at FZ for both time*real‐ sham.
**Figure S8a.** *Passive picture task ERP in relation to alcohol related pictures for real rTMS at T2 (straight line), real rTMS at (dash line), sham rTMS at T2 (dense dotted line) and sham rTMS at T0 for Pz.*
**Figure S8b.** *Passive picture task ERP in relation to non‐ alcohol related pictures for real rTMS at T2 (straight line), real rTMS at (dash line), sham rTMS at T2 (dense dotted line) and sham rTMS at T0 for Pz.*
**Figure S9.** *Repeated measures ANOVA for amplitude of P300 at electrode Pz at the three time points for alcohol related pictures*.
**Figure S10.** *Passive picture task ERP from real rTMS at T2 in relation to alcohol related images (straight line), real rTMS at T2 in relation to non‐alcohol images (dash line), sham rTMS at T2 in relation to alcohol related images (dense dotted line) and sham rTMS at T2 in relation to non‐alcohol images (sparse dotted line) for Cz.*
**Figure S11.** Bayes factor design analysis distribution plot of N with an estimated effect size of Cohens d of 0.55 aiming at a strong effect (boundary BF10 = 10).

## Data Availability

The data presented in this study are openly available in FigShare (doi 10.6084/m9.figshare.26770057).
