# Supplementary material for: No Effects of rTMS on Performance Monitoring and Attentional Bias in Patients With Alcohol Use Disorder: A Pilot Study
Source: Addict Biol. 2025 Nov 18;30(11):e70100. doi: 10.1111/adb.70100 (PMC12626745; doi:10.1111/adb.70100)

**Supplement**

**Table 1**

*Baseline Sample Characteristics*

| **Variables** | **rTMS + TAU**  **(*n* = 14)** | **TAU**  **(*n* = 16)** | **Total Sample**  **(*N* = 30)** |
| --- | --- | --- | --- |
| *Demographics* |  |  |  |
| Age, *M (SD)* | 48.9 (8.0) | 46.4 (10.0) | 47.6 (9.1) |
| Gender, *%male* | 100 | 94 | 97 |
| IQ score, *M (SD)* | 96.5 (14.8) | 98.2 (15.5) | 97.4 (15.0) |
| *AUD, M (SD)* |  |  |  |
| Age first ever alcohol use | 12.4 (3.8) | 13.3 (3.6) | 12.8 (3.6) |
| Years of problematic use | 16.3 (6.9) | 14.4 (7.9) | 15.3 (7.4) |
| Consumption alcohol *(gr/day)* | 133 (51) | 124 (62) | 128 (56) |
| Number previous treatments | 3.6 (1.4) | 4.7 (4.3) | 4.2 (3.2) |
| *Other substance use disorders* |  |  |  |
| Tobacco, *%* | 76 | 94 | 87 |
| Cannabis, *%* | 0 | 6 | 3 |
| Stimulants, *%* | 0 | 6 | 3 |
| Benzodiazepine, *%* | 0 | 6 | 3 |
| *Psychiatric comorbidity* |  |  |  |
| PTSD, *%* | 0 | 44 | 23 |
| Depression, *%* | 14 | 13 | 13 |
| OCD, *%* | 0 | 25 | 13 |
| Panic disorder, *%* | 0 | 0 | 0 |
| *Measurements baseline* |  |  |  |
| VAS, *M (SD)* | 0.9 (1.4) | 0.8 (1.2 | 0.9 (1.2) |
| OCDS, *M (SD)* | 7.3 (4.0) | 8.1 (3.1) | 7.7 (3.5) |
| AUQ, *M (SD)* | 15.9 (5.3) | 14.3 (5.9) | 15.1 (5.7) |
| Abstinent, *%* | 100 | 94 | 97 |
| Alcohol use (gr/day), *M (SD)* | 0 (0) | 0.13 (0.13) | 0.07 (0.03) |
| Heavy drinking, *M (SD)* | 0 (0) | 0 (0) | 0 (0) |
| *Use of medication* |  |  |  |
| Anticraving, *%* | 7 | 6 | 7 |
| Antidepressants, *%* | 14 | 50 | 33 |
| Antipsychotics, *%* | 14 | 44 | 30 |
| Benzodiazepines, *%* | 57 | 75 | 67 |

**Note:** M = Mean; SD = Standard Deviation; AUD = Alcohol Use Disorder; PTSD = Post-traumatic stress disorder; OCD = Obsessive compulsive disorder; VAS = Visual Analog Scale; OCDS = Obsessive Compulsive Drinking Scale; AUQ = Alcohol Urge Questionnaire

**Figure 1**

*Location of Fz, Cz, Pz of the EEG and rTMS target location on the right dLPFC*


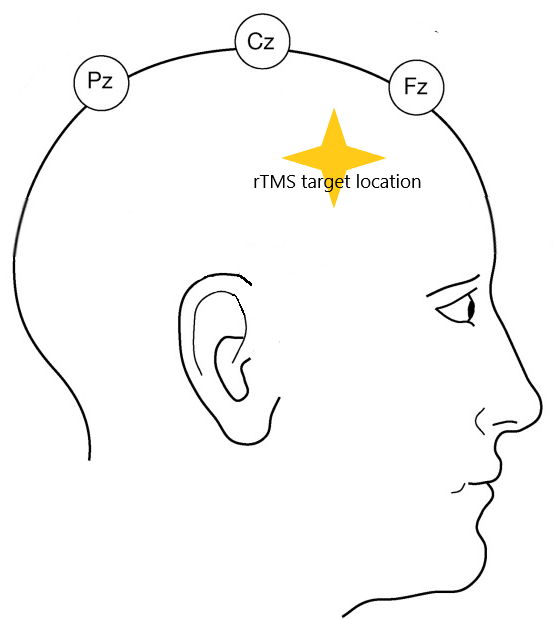


**Figure 2**

*Flanker task reaction times for correct responses (left graph) incorrect responses (middle graph) and too late responses (right graph) at start (T0), halfway (T1) and end (T2) of treatment.*


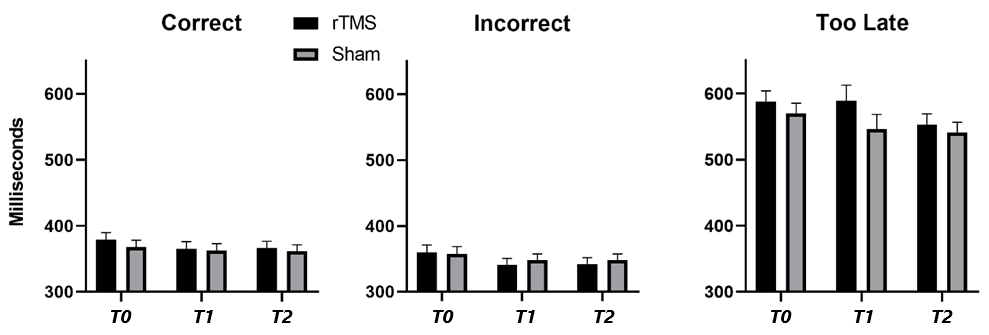


**Figure 3**

*Reaction accuracy for correct responses (left graph) incorrect responses (right graph) at start (T0), halfway (T1) and end (T2) of treatment.*


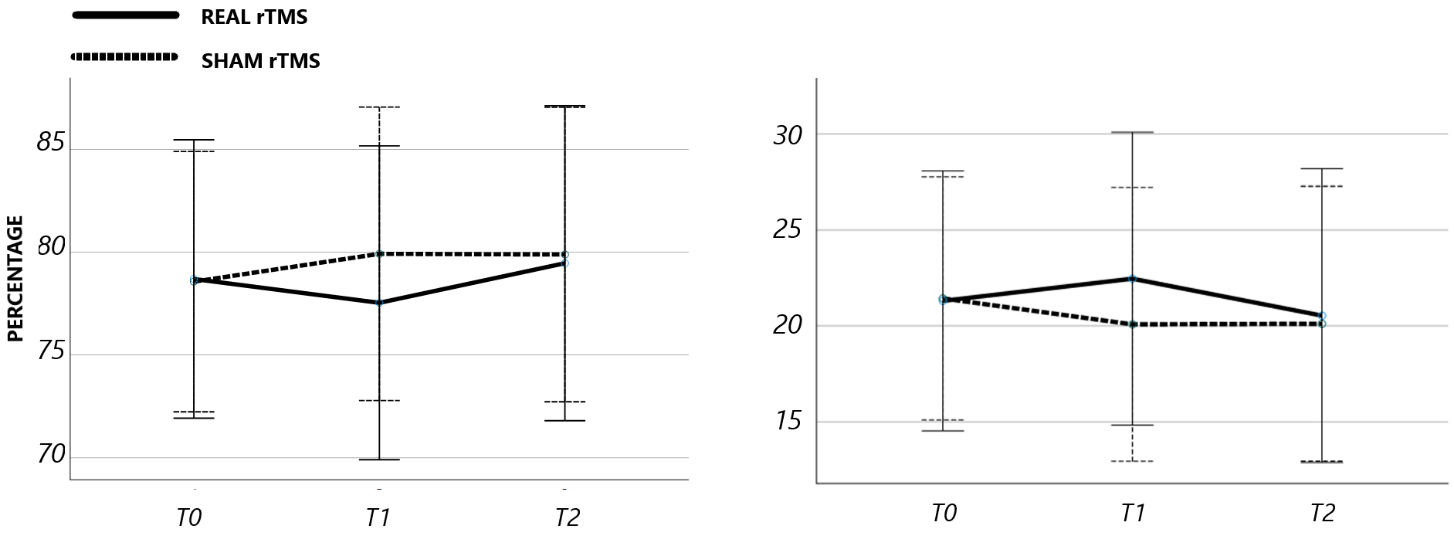


**Figure 4a**

*Flanker stimulus induced ERP from real rTMS at T2 (straight line), real rTMS at T0 (dash line), sham rTMS at T2 (dense dotted line) and sham rTMS at T0 (sparse dotted line) for FZ.*


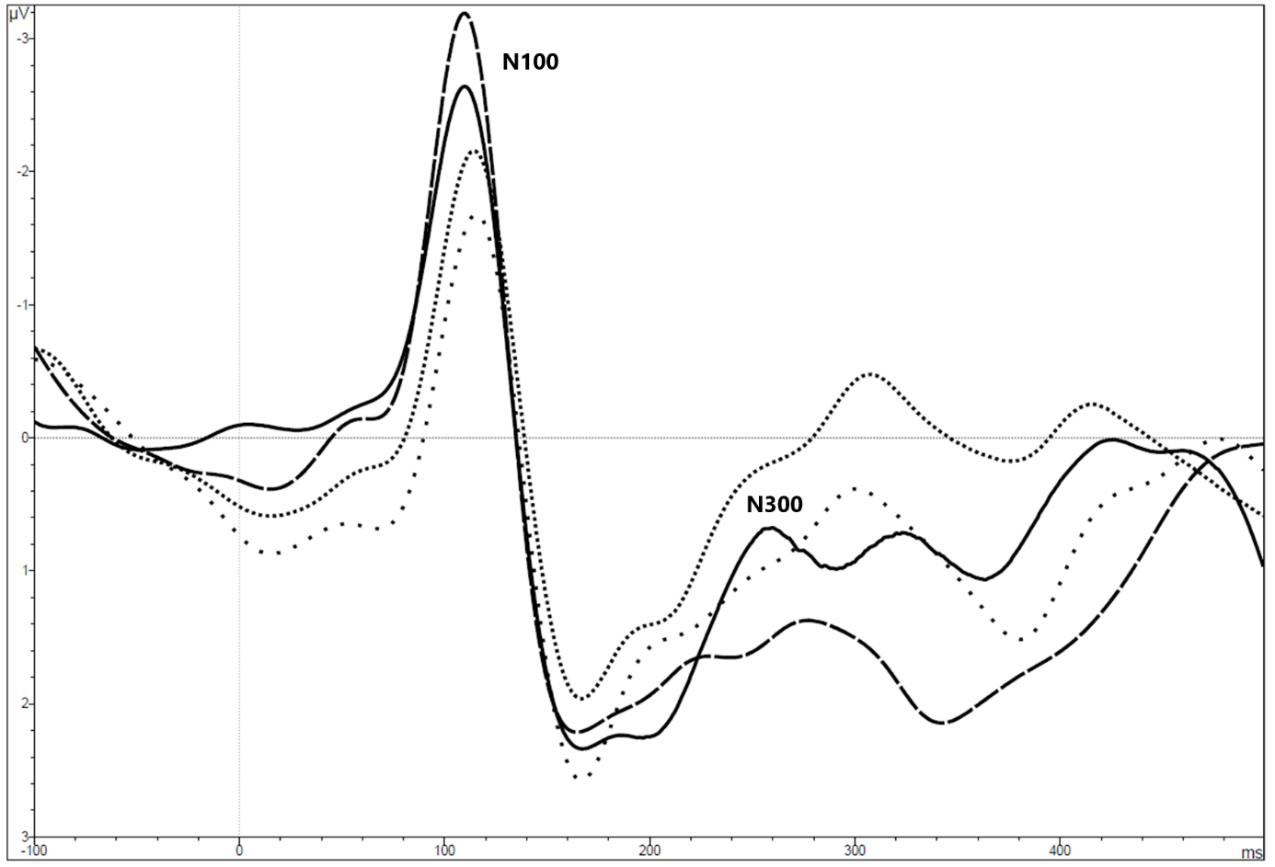


**Figure 4b**

*Flanker stimulus induced ERP from real rTMS at T2 (straight line), real rTMS at T0 (dash line), sham rTMS at T2 (dense dotted line) and sham rTMS at T0 (sparse dotted line) for CZ.*
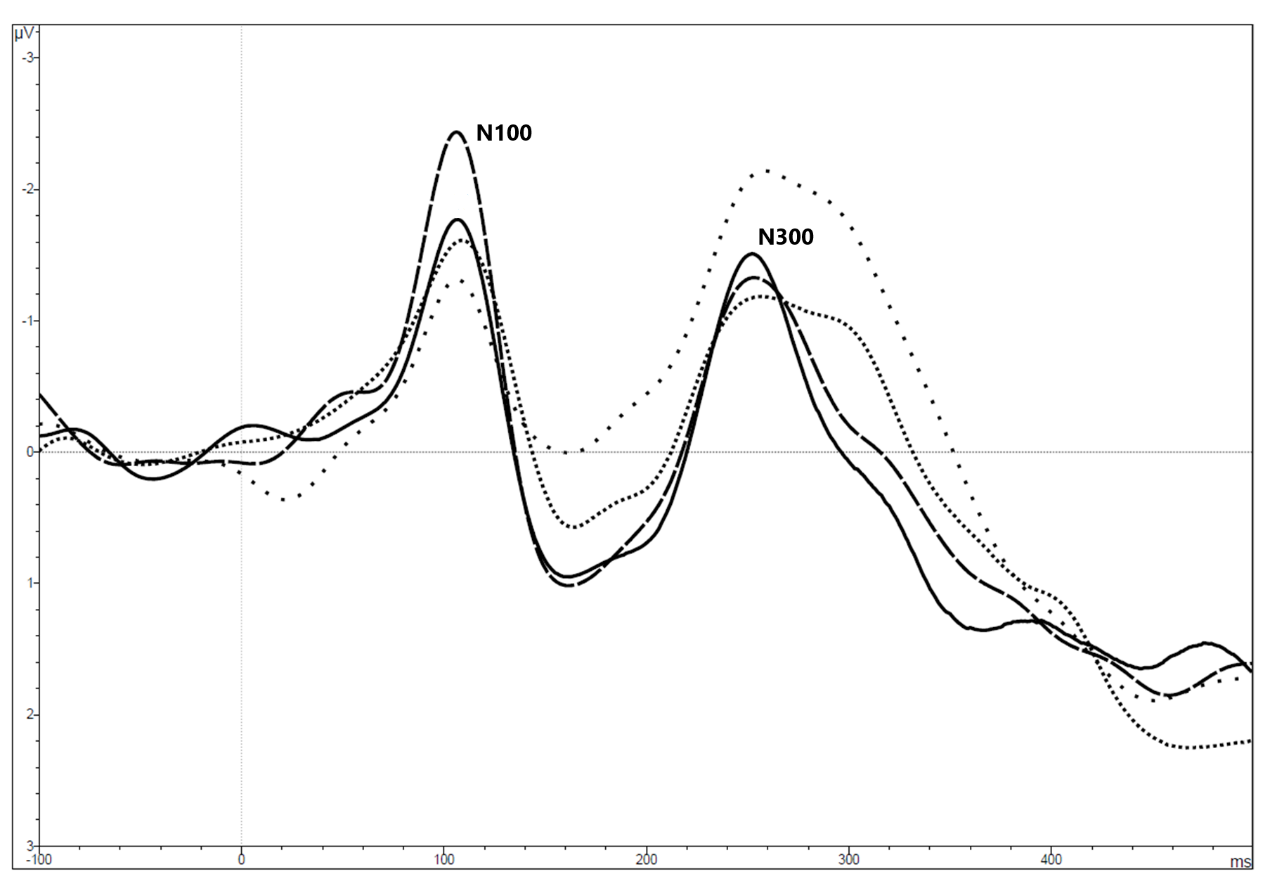


**Figure 4c**

*Flanker stimulus induced ERP from real rTMS at T2 (straight line), real rTMS at T0 (dash line), sham rTMS at T2 (dense dotted line) and sham rTMS at T0 (sparse dotted line) for PZ.*
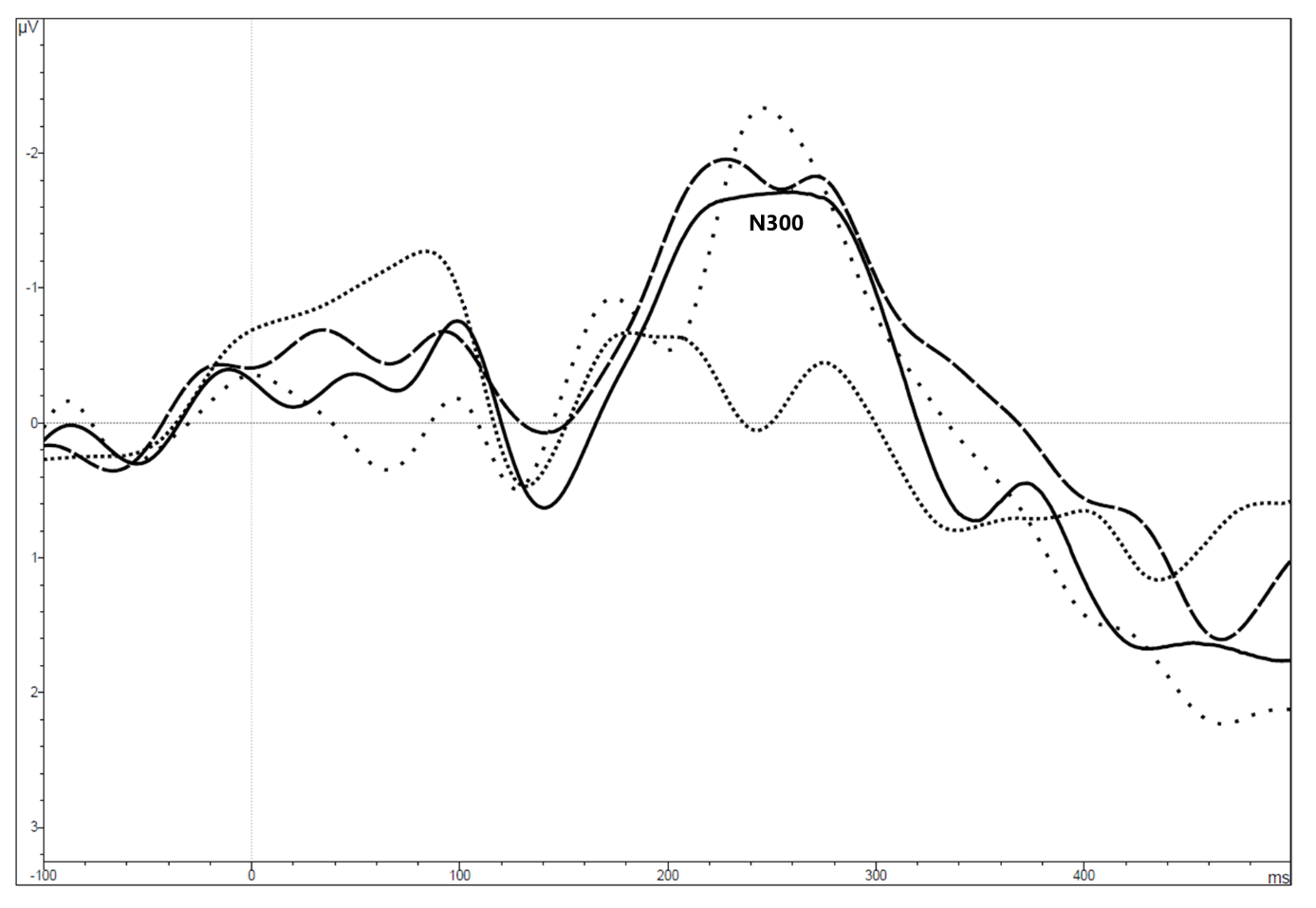


**Figure 3c**

*Flanker stimulus induced ERP from real rTMS at T2 (straight line), real rTMS at T0 (dash line), sham rTMS at T2 (dense dotted line) and sham rTMS at T0 (sparse dotted line) for PZ.*

**Figure 5**

*Repeated measures ANOVA N100 at electrode Fz at the three time points*

**Figure 6**

Bayesian plot of Flanker-stimulus locked N100 at FZ for both real and sham group.


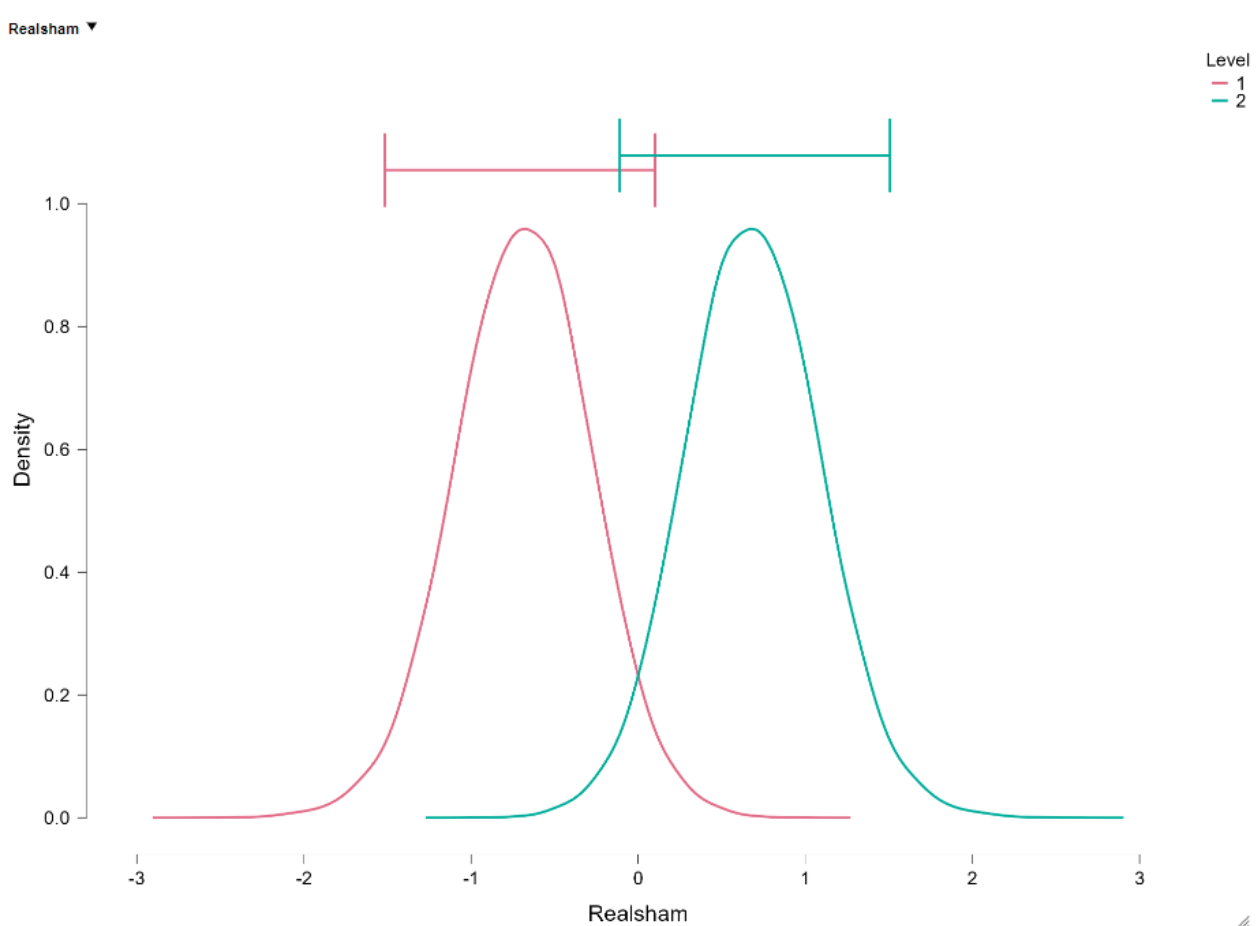


**Figure 7**

Bayesian plot of Flanker-stimulus locked N100 at FZ for both time*real- sham.


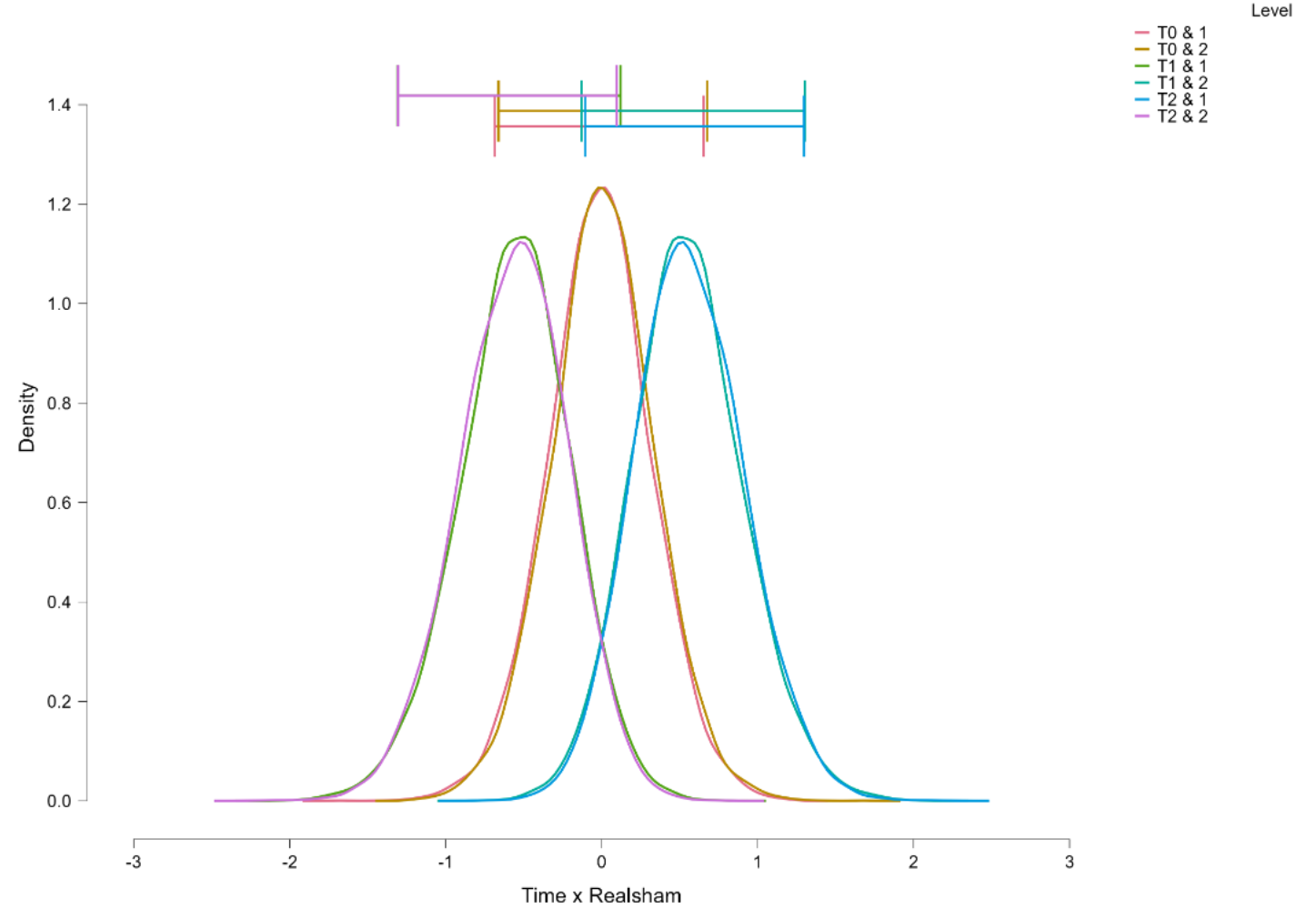


**Figure 8a**

*Passive picture task ERP in relation to alcohol related pictures for real rTMS at T2 (straight line), real rTMS at (dash line), sham rTMS at T2 (dense dotted line) and sham rTMS at T0 for Pz.*


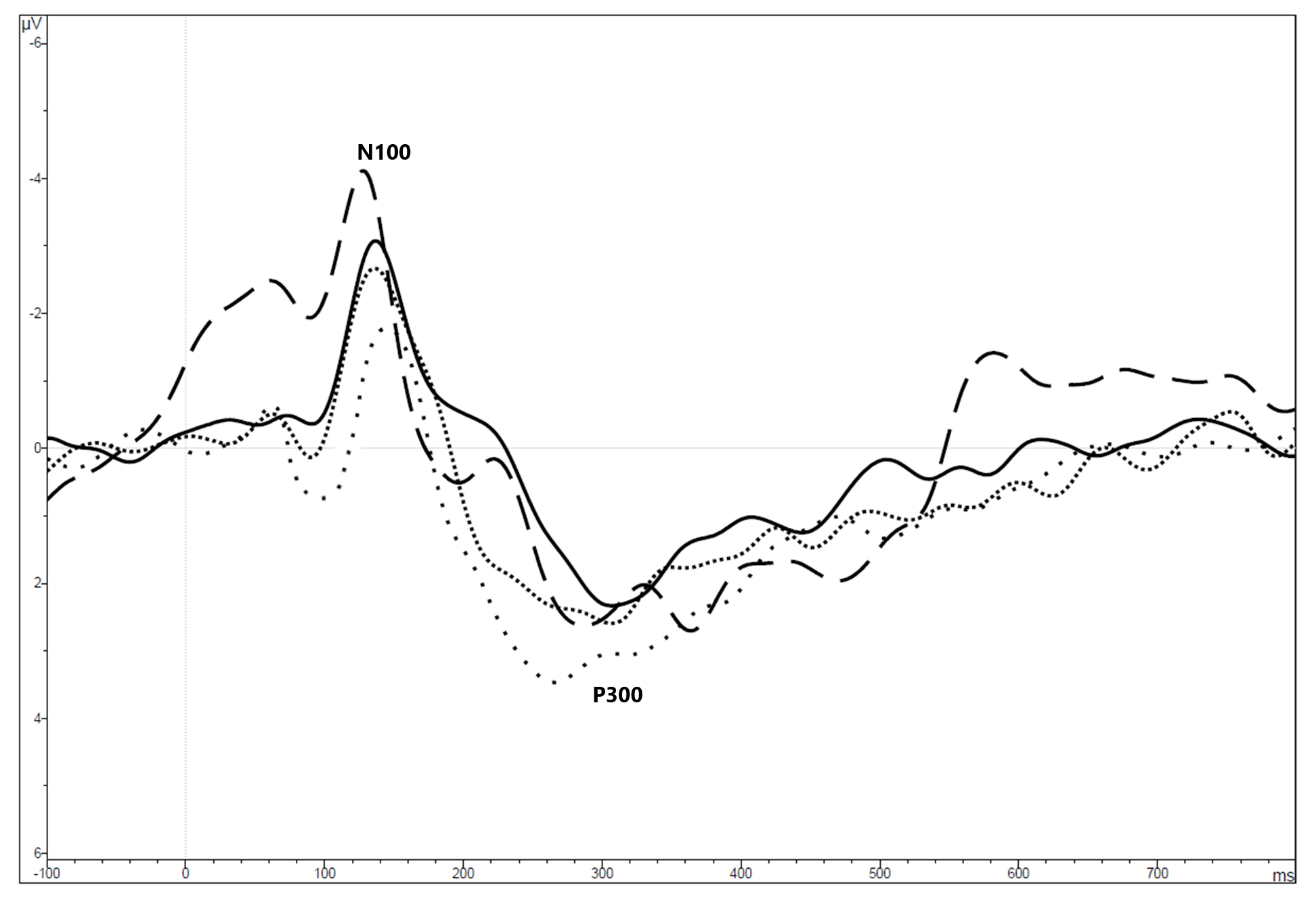


**Figure 8b**

*Passive picture task ERP in relation to non- alcohol related pictures for real rTMS at T2 (straight line), real rTMS at (dash line), sham rTMS at T2 (dense dotted line) and sham rTMS at T0 for Pz.*


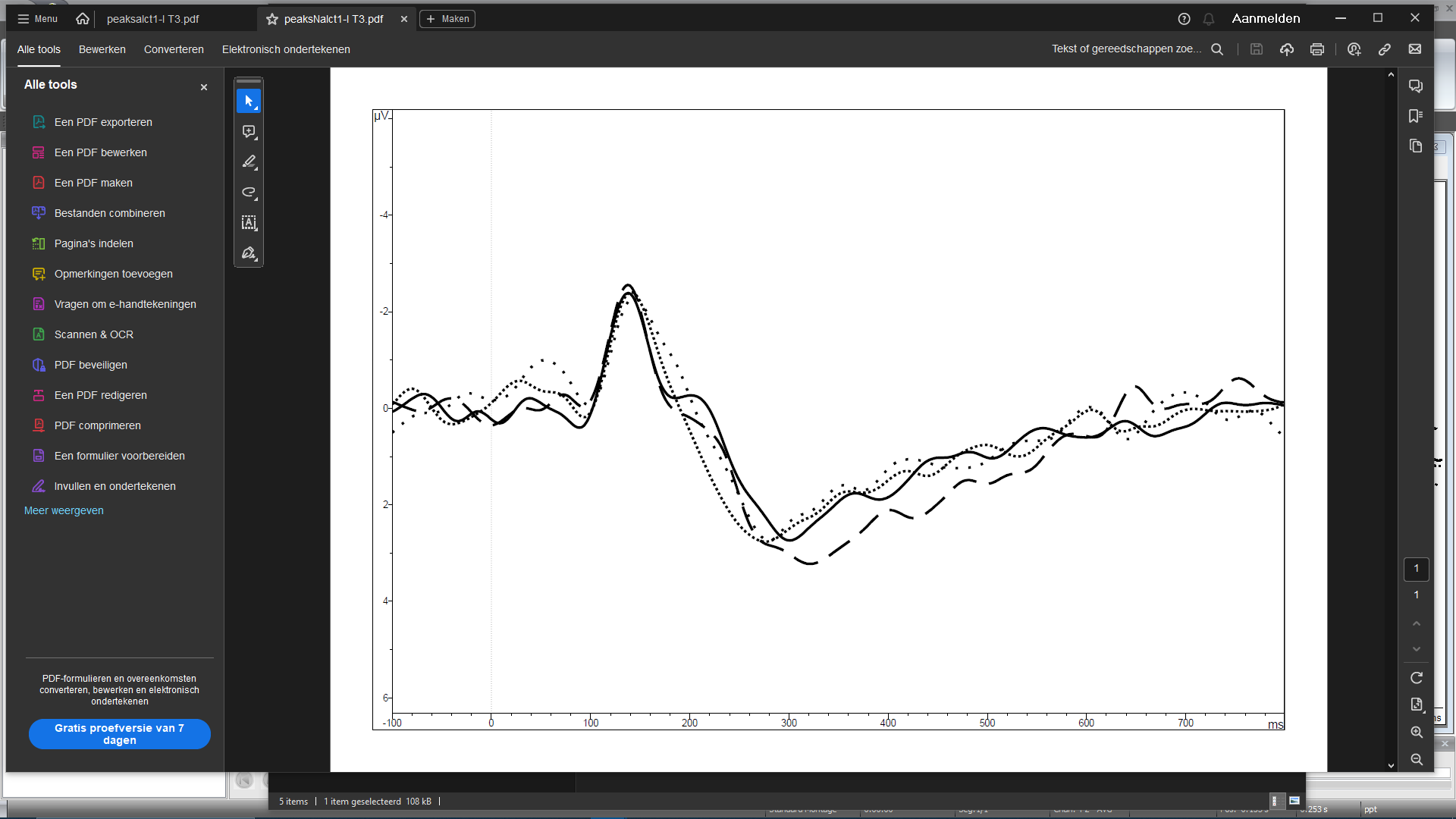


**Figure 9**

*Repeated measures ANOVA for amplitude of P300 at electrode Pz at the three time points for alcohol related pictures* ***
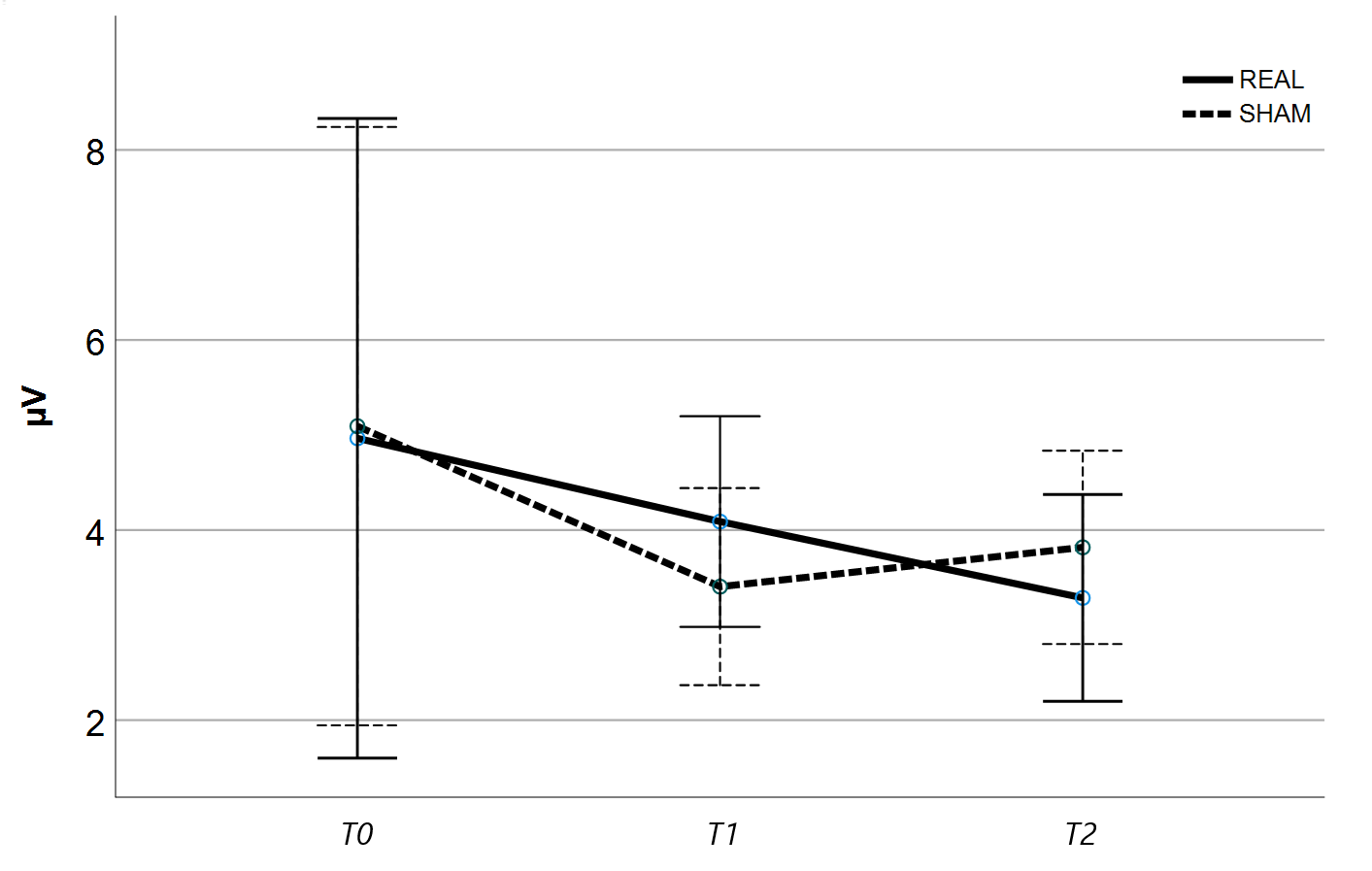
***

**Figure 10**

*Passive picture task ERP from real rTMS at T2 in relation to alcohol related images (straight line), real rTMS at T2 in relation to non-alcohol images (dash line), sham rTMS at T2 in relation to alcohol related images (dense dotted line) and sham rTMS at T2 in relation to non-alcohol images (sparse dotted line) for Cz.*


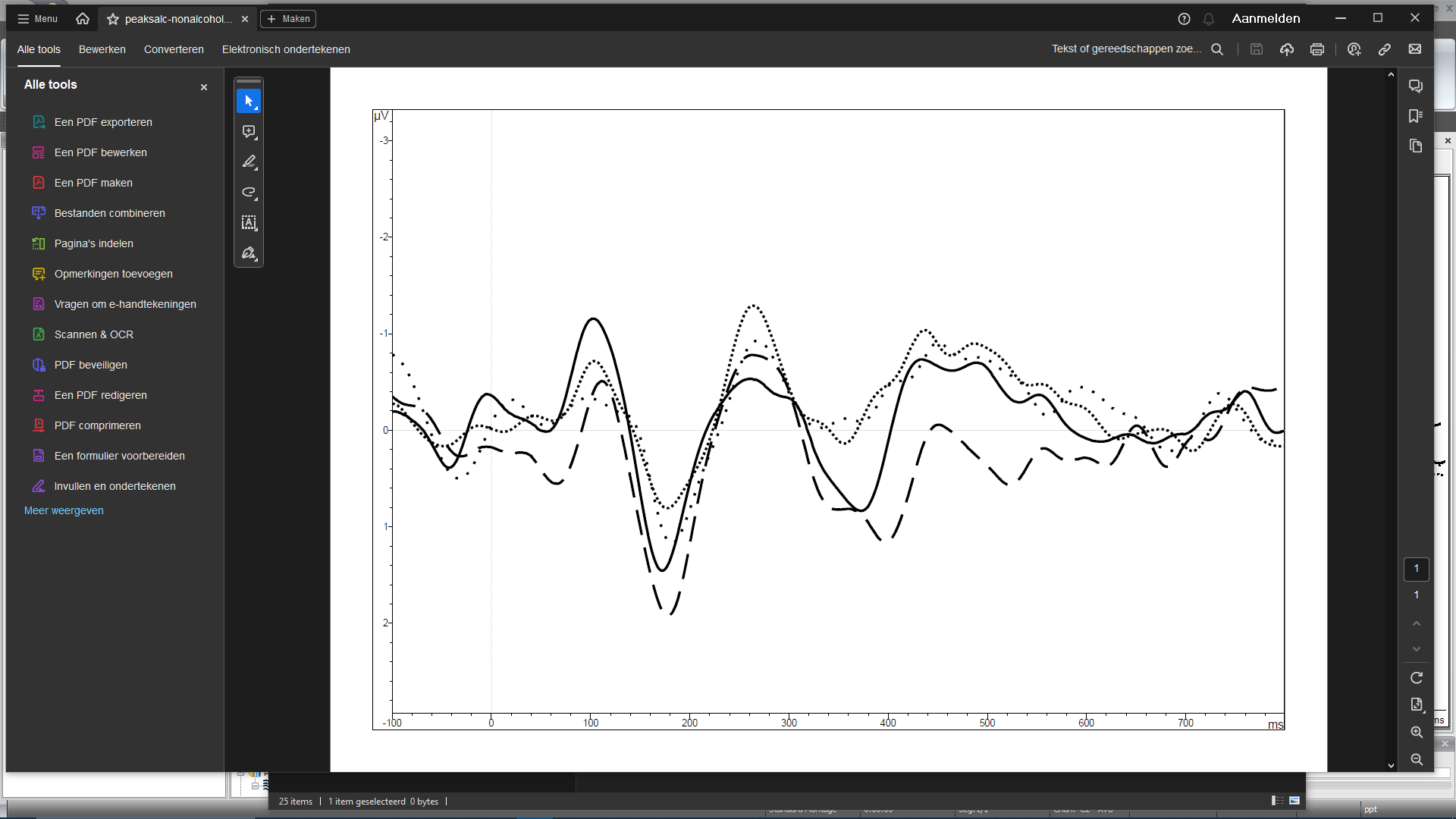


**Figure 11**

Bayes factor design analysis distribution plot of N with an estimated effect size of Cohens d of 0.55 aminig at a strong effect (boundary BF10=10 ).


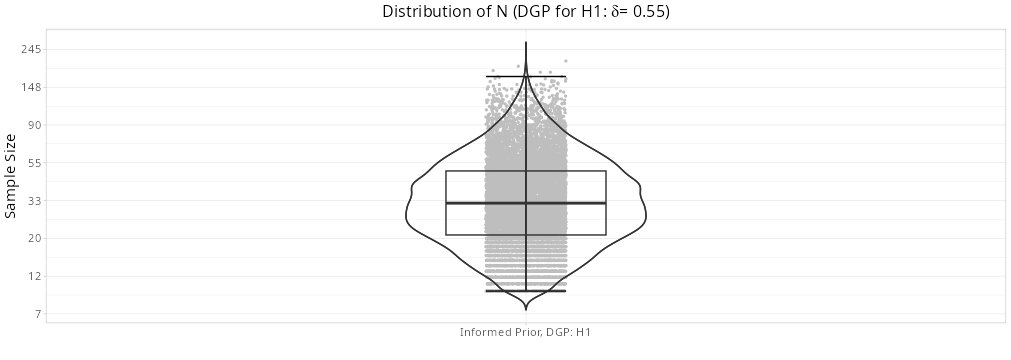

Supplement: Supplementary file 1 — Table S1 Baseline Sample Characteristics. Figure S1. Location of Fz, Cz, Pz of the EEG and rTMS target location on the right dLPFC. Figure S2. Flanker task reaction times for correct responses (left graph) incorrect responses (middle graph) and too late responses (right graph) at start (T0), halfway (T1) and end (T2) of treatment. Figure S3. Reaction accuracy for correct responses (left graph) incorrect responses (right graph) at start (T0), halfway (T1) and end (T2) of treatment. Figure S4a. Flanker stimulus induced ERP from real rTMS at T2 (straight line), real rTMS at T0 (dash line), sham rTMS at T2 (dense dotted line) and sham rTMS at T0 (sparse dotted line) for FZ. Figure S4b. Flanker stimulus induced ERP from real rTMS at T2 (straight line), real rTMS at T0 (dash line), sham rTMS at T2 (dense dotted line) and sham rTMS at T0 (sparse dotted line) for CZ. Figure S4c. Flanker stimulus induced ERP from real rTMS at T2 (straight line), real rTMS at T0 (dash line), sham rTMS at T2 (dense dotted line) and sham rTMS at T0 (sparse dotted line) for PZ. Figure S5. Repeated measures ANOVA N100 at electrode Fz at the three time points. Figure S6. Bayesian plot of Flanker‐stimulus locked N100 at FZ for both real and sham group. Figure S7. Bayesian plot of Flanker‐stimulus locked N100 at FZ for both time*real‐ sham. Figure S8a. Passive picture task ERP in relation to alcohol related pictures for real rTMS at T2 (straight line), real rTMS at (dash line), sham rTMS at T2 (dense dotted line) and sham rTMS at T0 for Pz. Figure S8b. Passive picture task ERP in relation to non‐ alcohol related pictures for real rTMS at T2 (straight line), real rTMS at (dash line), sham rTMS at T2 (dense dotted line) and sham rTMS at T0 for Pz. Figure S9. Repeated measures ANOVA for amplitude of P300 at electrode Pz at the three time points for alcohol related pictures. Figure S10. Passive picture task ERP from real rTMS at T2 in relation to alcohol related images (straight line), real rTMS at T2 i [file ADB-30-e70100-s001.docx]
